# Cost-efficient and Accurate Risk Assessment Instruments in Type 2 Diabetics with Greatest Risk for Cardiovascular Disease

**DOI:** 10.26502/fccm.92920485

**Published:** 2026-04-16

**Authors:** Meghana Kaipa, Devendra K. Agrawal

**Affiliations:** Department of Translational Research, Western University of Health Sciences, Pomona, California, USA

**Keywords:** ASCVD Risk Calculator, Cardiovascular Disease Risk, Clinical Decision-Making, Framingham Risk Score, Preventative Medicine, QRISK Model, Risk Prediction Models, Screening Tools, Type 2 Diabetes Mellitus, UKPDS Risk Engine

## Abstract

Cardiovascular disease remains a leading cause of death among individuals with Type 2 diabetes mellitus (T2DM). To better direct preventative care, many risk assessment tools have been developed to determine whether T2DM patients will develop cardiovascular disease. This paper analyzes three specific tools: UKPDS (United Kingdom Prospective Diabetes Study), the Framingham Risk Score, and the QRISK (QRESEARCH cardiovascular risk algorithm) calculator, with a focus on their predictive utility and limitations in clinical practice. All these models were designed using a variety of patient information, including demographics, cholesterol levels, and blood pressure values; however, each has its own drawbacks. The UKPDS is based on an outdated study and does not account for patients with a history of baseline heart disease. The Framingham Risk Score is less accurate when determining the risk of cardiovascular events in underserved populations, as the database it was developed from lacks sufficient representation of these groups. While the QRISK calculator incorporates additional patient factors to better address the gaps of previous tools, resulting in more accurate predictions, none of these tools are perfectly individualized. Patient data is constantly changing, highlighting the need for models that utilize machine learning algorithms. Such approaches allow for greater adaptability and can integrate data from biomarkers and continuous glucose monitoring devices. Improving the accessibility and implementation of these newer tools is essential to better address existing healthcare gaps and enhance preventative care in high-risk populations.

## Introduction

**Figure 1:**
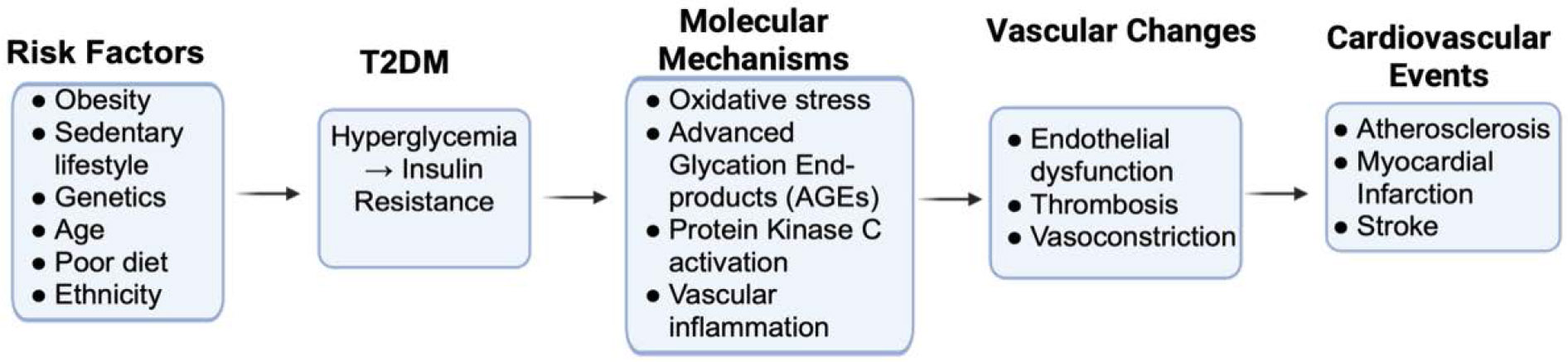
This flowchart illustrates how risk factors for T2DM can lead to molecular and vascular changes which result in cardiovascular events.

Type 2 Diabetes Mellitus (T2DM), the most common form of diabetes, occurs when the body cannot effectively use or produce insulin to break down blood glucose, resulting in high blood sugar. In 2021, approximately 38.4 million people (11.6% of the population) in the United States had diabetes [1-4], with that number only growing over the recent years. High blood glucose levels impact multiple organ systems in the body and can result in several issues when unmanaged, such as nerve and eye problems, kidney disease, pulmonary fibrosis, abdominal aortic aneurysm, infections, diabetic foot ulcer, inflammatory bowel disease, development of cardiovascular, musculoskeletal and other diseases [5-18]. Hyperglycemia, insulin resistance, and excess fatty acids, all of which occur in diabetics, increase oxidative stress, disrupting molecular signaling and increasing the production of products that can lead to vascular inflammation, vasoconstriction, thrombosis, and atherogenesis [19,20]. Figure 1 illustrates this general mechanism of how risk factors for T2DM may eventually result in cardiac events [21].

Cardiovascular Disease (CVD) is a common comorbidity affecting approximately 32.2% of individuals with T2DM [22]. As the leading global cause of death in the world, CVD remains a major cause of mortality for Type 2 diabetics. This necessitates careful evaluation, use of right tool for prognosis, preventive measures, risk management, develop better treatment and personalized management strategies [23-32].

There are currently many existing risk models which help predict whether Type 2 diabetics may develop CVD, but they are costly or lack precision in effectively identifying high-risk individuals. This paper will explore accurate, affordable tools for assessing CVD risk among T2DM patients. Developing or improving cost-effective risk assessment tools can enhance early identification and targeted prevention of CVD in T2DM populations.

## Methodology

A literature review was conducted to study cardiovascular risk assessment tools among individuals with Type 2 Diabetes Mellitus using comprehensive databases, including PubMed, Google Scholar, and relevant government health websites.

Keywords used to search the databases included: “Type 2 Diabetes,” “Cardiovascular Risk Assessment,” “UKPDS,” “Framingham Risk Score,” “QRISK,” and “Cost-effectiveness.” Most of the sources spanned the years 2017–2024, with the earliest source dating back to 1998 to better understand the UKPDS model, and the most recent source published in 2025. The variety of these sources helped establish the background of what these risk tools initially prioritized and how they have evolved to produce more individualized and accurate results, reflecting recent developments in clinical practice. Priority was given to peer-reviewed articles, with an emphasis on large systematic reviews that were more representative of diabetic populations.

## Evaluation of Current Risk Assessment Tools for Type 2 Diabetes Mellitus

The global burden of cardiovascular disease among patients with Type 2 Diabetes continues to grow, emphasizing the need for accurate risk-assessment models. While these tools intend to estimate cardiovascular risk among this population, their design, validation methods, and clinical applicability vary widely.

### UKPDS

The UK Prospective Diabetes Study (UKPDS) originally began in 1977 as a longitudinal study to determine which methods of treatment are better at managing T2DM such as diet therapy or intensive treatments like insulin [33]. Based on the study, a risk model was then developed to determine the likelihood of T2DM patients having atherosclerotic cardiovascular disease events based on several factors, including systolic blood pressure (SBP), HDL cholesterol values, smoking, and elevated HbA1c values.

It was an innovative tool when it first came out. Now it may not be the most feasible tool available, as the model was based on a study which is quite outdated. It was developed based on patients who did not have baseline heart disease or stroke at the time of their T2D diagnosis. Given how CVD is one of the most impactful comorbidities of T2DM, patients who are at risk for T2DM may also be at risk for CVD. A newer model, based on simulation studies, was created keeping this in mind, predicting secondary MI and stroke events among T2DM patients [34]. Since it has not been externally validated, the UKPDS model may not be the best prediction tool for current populations.

### Framingham Risk Score

One of the features of the UKPDS model was that it was based on a variety of factors that may contribute to the development of CVD. Other models, like the Framingham Risk Score (FRS), differ in what they use as their basis. Specifically, the FRS considers the biological sex differences that contribute to CVD risk. Like other models, it still plugs in key personal health data (age, sex, total and HDL cholesterol, SBP, smoking status, and diabetes status). Each factor is then assigned points, which are summed to determine which category the patient falls into, estimating the 10-year risk of having a cardiovascular event. The risk score ranges from −3 to 21+, with different scoring rubrics based on the patient’s sex. The three categories include low (<13 in women, < 11 in men; risk <10%), moderate (13-17 in women, 11-14 in men; risk 10-20%), and high (> 17 in women, >14 in men; risk ≥20%) [35-37].

In 2009, a new set of guidelines was published, enhancing the algorithm. By incorporating factors such as a family history of coronary artery disease in a first-degree relative with a first event before age 60, as well as the evaluation of high-sensitivity C-reactive protein (hs-CRP) levels, a marker used to predict cardiovascular events in patients without prior CVD, the model’s validity improved [35,38].

Despite these updates, the Framingham Risk Score’s role in contemporary practice highlights important limitations that must be acknowledged when applying it to diverse patient populations. While it is an ongoing project, it is important to address some of the key benefits and drawbacks of this risk assessment model. The predictors are clinically relevant and practical; however, its accuracy might be limited in certain patient populations. For underserved populations, there was not as much data when creating the model, hence the lack of accuracy currently. This may guide physicians towards the wrong treatment as it has the potential to under-/over-estimate risk for patients [39], which could lead to detrimental effects.

### QRISK

Based on the limitations of older tools like the Framingham Risk Score, a newer model like the QRISK calculator was developed to target and patch some of those gaps. It still considers the traditional factors (age, sex, smoking, cholesterol levels), but importantly also includes newer ones like ethnicity, treated hypertension, chronic kidney disease, atrial fibrillation, rheumatoid arthritis, severe mental illness, migraines, etc. [40]. By adding more factors, the model can better paint a picture of the patient and produce a more accurate risk outcome. It produces a percentage to determine risk of CVD events like MI or stroke over the next 10 years, assuming the patient does not already have CVD. A score >10% indicates a moderate-high risk and is beneficial for the physician to know the intensity of treatment they may need to prescribe to their patient.

The model is consistently being updated, with the QRISK-3 currently in practice. It is calibrated based on age, ethnicity, and baseline disease status. The accuracy is also improving as new patient population factors are added to the risk assessment algorithm, such as pregnancy in women and erectile dysfunction in men [40, 41]. As it addresses the drawbacks of previous risk assessment tools and grows, the results of the QRISK seem promising in a variety of patient populations.

## Limitations of Current Risk Assessment Tools

Three tools have been reported and evaluated for their potential to identify the risk of developing CVD in patients with T2DM: UKPDS, Framingham Risk Score, and QRISK. The main limitation of UKPDS was that it did not consider patients with baseline heart disease at the time of T2DM diagnosis. Even though it was updated to include that factor, the new tool was based on simulation studies which was not as accurate compared to real-world clinical data.

The Framingham Risk Score included a much higher number of predictors which makes the model more accurate, though it was not developed with a lot of information from underrepresented populations. Seeing as how this assessment tool was crucial in effecting treatment plans that physicians would provide, a lot of patients from those populations were often underestimated in terms of their risk levels. This contrasts with other populations that were more highly represented, who often had overestimated risk scores. The population bias can negatively influence treatment plans, which would be harmful for patients.

The QRISK has advanced quite a bit from the moment of development, where it now incorporates a wider variety of factors (including baseline diseases) and a larger number of people as basis for model calibration. The wider array of populations included in the study, makes this a far more appropriate and accurate tool in determining CVD risk among Type 2 diabetics.

There are a couple of drawbacks for all these tools. Firstly, none of these models are individualized for the specific patient. While these approaches can get close by including the cross-section of several different factors, there is a lot that can affect glucose levels, hence the severity of T2DM. Additionally, many patients' treatment plans include a combination of lifestyle (diet, exercise) and medications, which these models do not take into consideration. Table 1 summarizes the benefits and limitations of the three specific risk assessment tools as discussed above.

Continuous glucose monitors (CGM) are becoming much more common for diabetic patients because it helps track their glycemic variability (fluctuations in blood glucose levels) [42]. It can help them better understand their disease and factors that may affect their glucose levels (such as sleep, certain foods, exercise, etc.). Although CGMs are much less accessible in diabetic patients in developing nations, this is still one of the best tools patients have in tracking the progression of diabetes, as they can see the effects of their actions in real-time.

## Emerging Approaches

The perfect risk assessment tool would be cost-friendly, highly accessible, accurate, and personalized to the patient’s specific needs. Companies have been trying their best in creating models that cover these bases, however while they target one or two of these, they still have not succeeded in covering them all. Incremental advances, such as incorporating machine learning (ML) approaches that integrate biomarkers, patient-specific clinical data, and information derived from continuous glucose monitors, may offer a practical pathway toward improving the accuracy and personalization of future risk assessment tools [43].

**Figure 2:**
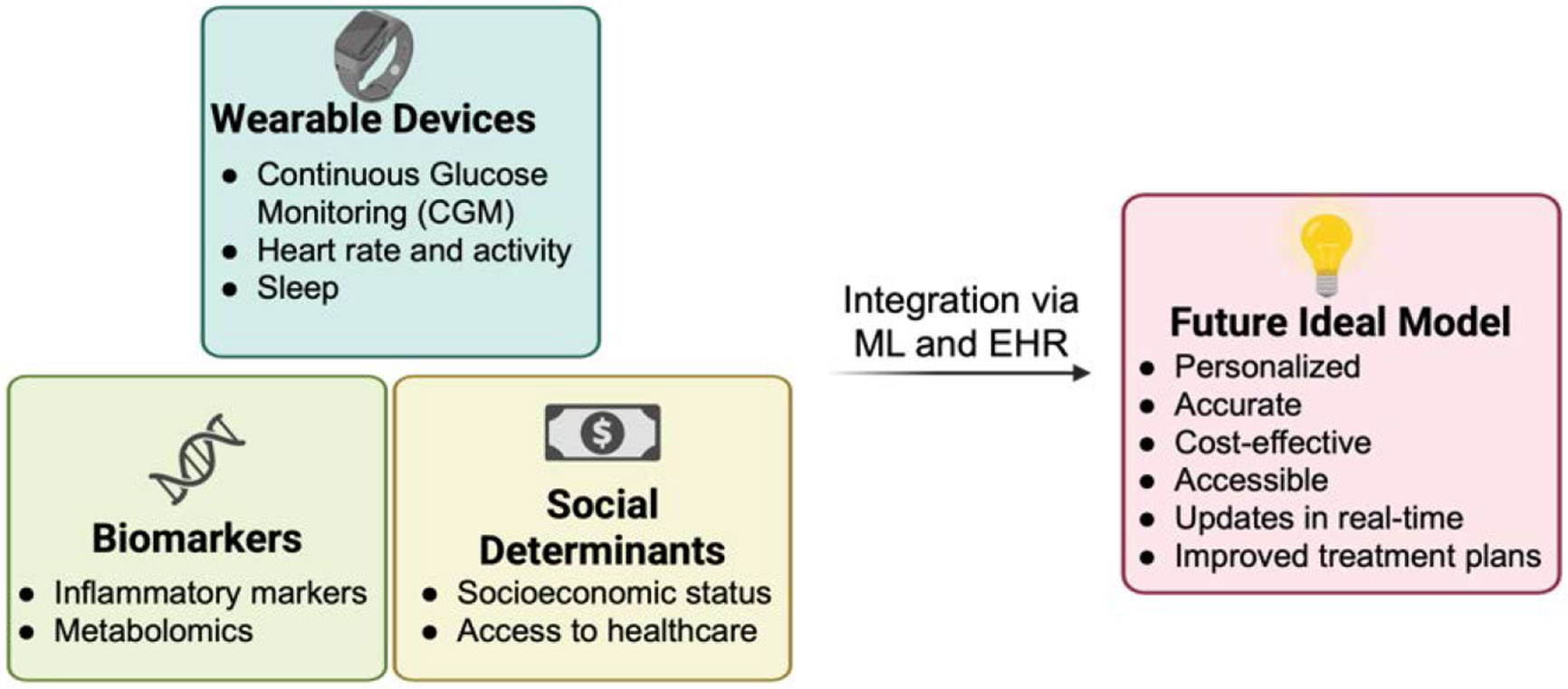
Conceptual framework illustrating the integration of emerging data sources such as wearable devices, biomarkers, and social determinants of health into ML-based models to improve personalization, accuracy, cost-effectiveness, and accessibility of risk assessment tools. EHR, electronic health record; ML, machine learning.

These new models can identify complex relationships between variables which current models may overlook. Biomarkers from inflammatory profiles or other metabolomic panels, along with wearable device data such as heart rate, activity and sleep duration can enhance predictive accuracy. Additionally, integrating social determinants of health such as socioeconomic status and access to care can address risk disparities underserved populations in healthcare currently face. Assimilation of this data with electronic health records (EHR) eases clinical decision making as all relevant data is readily available and can result in more timely and accurate treatment advice. Figure 2 depicts a conceptual framework highlighting the benefits of these emerging approaches. However, current issues with the privacy of health data and unequal access to technology (which a lot of data points may depend on) remain and need to be considered when implementing these approaches.

## Discussion

Metabolomics and proteomics provide a lot of insight when combined with high-throughput technologies to determine which biomarkers may be indicative of the development of CVD [44]. By targeting specific factors in the body in addition to the traditional metrics, a much more accurate assessment can be performed. The current effective tools include population-specific considerations such as ethnicity and socioeconomic factors which provide a much more holistic approach to understanding disease. If these new risk assessment tools get incorporated within healthcare systems, primary care physicians would be much more informed about the specifics about their patient’s diagnosis, and it would be crucial in providing effective preventative care. Additionally, these suggestions and treatment plans could be made through telehealth software, which would make it more accessible to the populations who may not receive healthcare as frequently [45].

## Implications

With the regular implementation of improved risk assessment models, preventative care would grow tremendously. Not only would individualize treatments better ensure efficacy through directly targeted measures, but it would help physicians map out how they can best serve their patients. These tools could guide the intensity of their treatment and how much lifestyle interventions can help Type 2 diabetics.

Additionally, developing cost-effective and accurate tools could advance public health initiatives. Since medication doses would be much more precise, it would potentially reduce the burden of CVD healthcare costs, as less patients would develop severe CVD. Not only would this lower mortality rates, but it would help save hospitals and patients from a large financial burden.

In the future, these models should be implemented in healthcare systems worldwide to expand access to personalized healthcare. Additionally, obtaining a larger percentage of data from wearable technology and using AI for risk prediction could be the future of preventative medicine for Type 2 diabetics.

## Conclusion

Accuracy and affordability must be delicately balanced when developing new healthcare tools. Often when one is focused on, the other becomes overlooked. Historically, this balance has been difficult to achieve in CVD risk assessment for individuals with Type 2 Diabetes [46]. Three tools that aimed to address this problem were the UKPDS, Framingham Risk Score, and QRISK. They all incorporated several predictors ranging from mostly biological to some environmental, with the variety of factors increasing respectively. These models were not as effective when looking at underserved populations (mainly because there was not enough data collected from these groups), which led to inaccurate results. The algorithms could also be more personalized by including data from continuous glucose monitors and specific biomarkers from metabolomics and proteomics studies which specifically look at biomarkers that are indicative of increased risk of CVD. Future research could focus on developing ML algorithms that can incorporate personalized data for increased accuracy and efficacy. Additionally, integrating these models into healthcare systems increases accessibility and changes how physicians can approach preventative care. Treatment plans would become optimized with specific approaches that can prevent CVD event risk in the future. This can help from a public health perspective too when considering the financial burden CVD patients have on the healthcare system. Ultimately, developing cost-effective, personalized, and widely accessible risk assessment tools has the potential to reduce cardiovascular disease burden in individuals with Type 2 Diabetes while advancing equitable, preventative care across diverse populations.

## Figures and Tables

**Table 1:** Benefits and limitations of three specific cardiovascular risk assessment tools for patients with T2DM: UKPDS, Framingham Risk Score, and QRISK.

Risk Assessment Tool	Benefits	Limitations
**UKPDS**	Based on a large longitudinal study Specifically developed for patients with Type 2 diabetes Incorporates clinically relevant factors (SBP, HDL, smoking, HbA1c)	Based on an outdated study (1977 cohort) Excludes patients with baseline heart disease or stroke Newer model based on simulation studies lacks external validation May not be applicable to current, diverse populations
**Framingham Risk Score (FRS)**	Incorporates sex-specific differences in CVD risk Provides clear 10-year risk stratification (low, moderate, high) Updated guidelines include family history and hs-CRP Uses a variety of clinically relevant and practical predictors	Limited accuracy in underserved populations due to lack of representation in original dataset May under- or over-estimate risk in certain groups Potential to guide inappropriate treatment decisions
**QRISK**	Incorporates a wider range of patient factors (ethnicity, comorbidities, socioeconomic-related variables) More accurate risk prediction across diverse populations Continuously updated (e.g., QRISK-3) Provides individualized 10-year risk percentage to guide treatment intensity	Still not fully individualized despite expanded variables Requires more extensive patient data Designed for primary prevention, not applicable for patients with pre-existing CVD

CVD, cardiovascular disease; HDL, high density lipoprotein; hs-CRP, high sensitivity C-reactive protein; SBP, systolic blood pressure.

## Key Points

- Type 2 Diabetes Mellitus (T2DM) significantly increases cardiovascular disease (CVD) risk, affecting over 30% of individuals with T2DM and contributing substantially to global morbidity and mortality.
- Existing CVD risk assessment tools for T2DM aim to guide prevention, but many lack sufficient accuracy, affordability, or applicability across diverse populations.
- The UKPDS risk model, though historically important, is limited by outdated data and reduced relevance to modern T2DM populations, particularly those with baseline cardiovascular disease.
- The Framingham Risk Score incorporates multiple biological predictors and sex-based differences, but its limited representation of underserved populations can lead to risk misclassification and inequitable care.
- The QRISK model addresses several limitations of earlier tools by incorporating ethnicity, comorbidities, and socioeconomic factors, making it more accurate and generalizable for diverse patient populations.
- Despite improvements, current models remain population-based rather than fully individualized, failing to account for real-time glycemic variability, lifestyle interventions, and treatment regimens.
- Emerging technologies such as continuous glucose monitoring (CGM), metabolomics, and proteomics offer opportunities to enhance personalization and predictive accuracy of CVD risk assessment.
- Machine learning–based models integrating clinical data, biomarkers, and wearable technology represent a promising direction for developing cost-effective and precise risk stratification tools.
- Implementing improved, accessible risk assessment tools within healthcare systems could enhance preventative care, reduce CVD burden, and lower long-term healthcare costs, particularly for underserved populations.
